# Lactic Acid Bacteria as Potential Biocontrol Agents for Fusarium Head Blight Disease of Spring Barley

**DOI:** 10.3389/fmicb.2022.912632

**Published:** 2022-07-22

**Authors:** Micheal B. Byrne, Ganesh Thapa, FIona M. Doohan, James I. Burke

**Affiliations:** ^1^School of Agriculture and Food Science, University College Dublin, Dublin, Ireland; ^2^School of Biology and Environmental Science, University College Dublin, Dublin, Ireland

**Keywords:** biological control, *Fusarium* head blight, lactic acid bacteria, barley (*Hordeum vulgare* L.), *pathogenesis-related* (PR) genes

## Abstract

Fusarium head blight (FHB) is a devastating disease encountered by spring-grown barley. Traditionally, synthetic chemicals have been used to control this disease on small grain cereals. A move toward biological control agents as part of sustainable agriculture is pertinent due to the evolutionary mechanisms employed by fungal diseases to circumvent current protection strategies. This study evaluated the effect of six lactic acid bacteria isolates on the development of FHB under *in vitro* and glasshouse conditions. The relative expression of *Fusarium* marker genes and transcription factors under *Fusarium* infection was examined. Dual-culture assays observed inhibition zones of up to 10 and 17% of total plate area for *L. amylovorus* FST 2.11 and *L. brevis* R2Δ, respectively. Detached leaf assays validated the antifungal activity and showed the potential of all test isolates to significantly inhibit sporulation of *Fusarium culmorum* and *Fusarium graminearum* strains. Spray inoculation of lactic acid bacteria to barley spikelets prior to *Fusarium* spore application significantly reduced disease severity for five candidates (*P* < 0.05) under glasshouse conditions. Mycotoxin analysis revealed the ability of *L. amylovorus* DSM20552 to significantly reduce deoxynivalenol content in spikelets (*P* < 0.05). A preliminary gene expression study showed the positive influence of lactic acid bacteria on the expression of important defense-related marker genes and transcription factors upon FHB. These results indicate the potential of lactic acid bacteria to be included as part of an integrated pest management strategy for the management of FHB disease. This strategy will reduce FHB severity and deoxynivalenol (DON) contamination of spring barley, leading to high acceptance in the grain market.

## Introduction

Barley (*Hordeum vulgare* L.) is among the world’s first domesticated land plants that has underpinned the development of present-day civilizations. It is one of the most important food staples considered to be the premium grain used by malting and brewing industries (Ullrich, 2010; CGIAR, 2012; Murphy, 2016). The Irish brewing and distilling sector has shown a growth rate of 4% between 2014 and 2019 with a 22% increase in the number of micro-breweries. The number of distilleries has increased to 38 in 2020 with global sales at 20 million nine-liter cases (Umego and Barry-Ryan, 2022). Therefore, it is imperative that we provide an active concern for world barley production and the threats associated with the escalation of crop losses as we enter an era of uncertainty surrounding food security. FHB is a serious spoilage disease encountered by barley, wheat (*Triticum aestivum*), and other small grain cereals worldwide (McMullen et al., 1997; Wegulo et al., 2015; Gunupuru et al., 2018). Primary agents of the disease vary across geographical regions with *Fusarium graminearum*, *Fusarium culmorum*, *Fusarium poae*, and *Fusarium avenaceum* encountered by European cereal growers (Bottalico and Perrone, 2002; Wegulo et al., 2015; Beccari et al., 2017). *Fusarium* species are also the causal organism of *Fusarium* foot rot and *Fusarium* seedling blight of small grain cereals resulting in reduced germination and poor plant establishment leading to intensified yield and quality losses (Brennan et al., 2003; Goswami and Kistler, 2004; Khan et al., 2006; Scherm et al., 2013). Accordingly, barley’s worth as a seed source for malt production for brewing and other food industries declines (Parry et al., 1995; Scherm et al., 2013; Hückelhoven et al., 2018). In barley, Fusaria of the FHB complex secure our attention as they produce a wide range of mycotoxins including: DON, HT-2 toxin, T-2 toxin, aflatoxin, and ochratoxin, known instigators of chronic and acute mycotoxicosis in humans and animals (Placinta et al., 1999; Haidukowski et al., 2005; Nielsen et al., 2014; Bolechová et al., 2015). The consequence of extreme disease outbreaks resulting in an FHB epidemic has been seen with extensive economic and sociological impacts (Jones, 2000; McMullen et al., 2012).

To circumvent additional costs associated with reduced yield, poorer quality, and extra costs of cleaning grain, synthetic fungicides have traditionally been applied to prevent FHB disease outbreaks (Smiley et al., 2005; Blandino et al., 2012; Matny, 2015). However, the efficacy of available fungicides is uncertain due to the evolving nature of *Fusarium*, changing weather patterns, and the inherent variability of cultivar resistance (Simpson et al., 2001; Magan et al., 2002; Blandino et al., 2012; Talas and McDonald, 2015). Even with the introduction of disease predictive models allowing for optimal timing of fungicide applications, their employment only remains practical for a one-off application at mid-anthesis, with further treatment costs failing to be justified by yield and quality improvements (McMullen et al., 1997; D’Angelo et al., 2014). Extensive field studies on azole-based fungicides to manage FHB and mycotoxin accumulation presented reductions between 40 and 70%, respectively (Paul et al., 2008, 2018; Willyerd et al., 2012; D’Angelo et al., 2014). However, with increscent fungicide applications, over time, gradual resistance among the target organisms is a real possibility (Ferrigo et al., 2016). Notably, the application of the fungicide azoxystrobin 2 days post-artificial inoculation of wheat spikelets with *Fusarium* spp. resulted in enhanced DON production per unit of pathogen (Simpson et al., 2001). Another study quantified that sub-lethal levels of prothioconazole increased DON production in wheat (Audenaert et al., 2010). Consequently, there has been a renewed focus on the implementation of integrated pest management (IPM) systems as both an economically and ecologically justified means to reduce the risks to human health and the environment (Union, 2009; Pretty and Bharucha, 2015; Cowger et al., 2016).

To date, research has focused on the development of proficient biological control agents (BCAs), with the aim of contriving new commercially applicable FHB antagonists (Wachowska and Głowacka, 2014; Xue et al., 2014; Wang et al., 2015). A potential group of 22 endophytes to control *F. graminearum* (30–51% inhibition) and *F. culmorum* (15–53% inhibition) *in vitro*, including the discovery of three novel endophytic species, i.e., *Aureobasidium proteae, Phoma glomeramycota* and *Sarocladium kiliense*, have been reported (Comby et al., 2017). Recently, the mode of action of the known antifungal species *Bacillus amyloliquefaciens* Y1 has been identified as the antifungal compound cyclo(D-PRO-L-VAL), which was characterized using H1 and C13 nuclear magnetic resonance techniques (Jamal et al., 2017). Furthermore, it has been shown that detoxification of mycotoxin-infused produce, including grain, is possible through the addition of biological agents prior to and during the processing of foodstuffs (Petchkongkaew et al., 2008; Tian et al., 2016). Field trials gave comparable results for reductions in DON accumulation and FHB severity following applications of *Bacillus* subtilis strain RC218 (Palazzini et al., 2016; Cantoro et al., 2021). Several strains of lactic acid bacteria (LAB) have similarly shown promise through their enhancement of organoleptic properties and ability to mitigate fungal contamination of foodstuffs, most notably their incorporation into starter cultures during malting and the formulation of probiotics (Schnürer and Magnusson, 2005; Kedia et al., 2007; Franco et al., 2011; Rathore et al., 2012; Nakkarach and Withayagiat, 2018). The versatile use potential was further confirmed to reduce mycotoxin accumulation and gushing when applied to barley *in situ* (Lowe and Arendt, 2004; Peyer et al., 2016). It is considered that their role in fungal inhibition is through the production of organic acids, proteinaceous compounds, and other low molecular weight compounds (Lynch et al., 2016; Siedler et al., 2019). Despite making major progress, the role of many BCAs in the regulation of plant defense responses, mechanistic of LAB-mediated resilience to biotic stresses, viz., FHB disease of barley, remains limited (Miedaner et al., 2018; Gómez-Lama Cabanás et al., 2021).

As a pathogen-associated molecular pattern (PAMP) is conserved molecules among pathogens and PAMP-triggered immunity (PTI) contributes to basal and non-host resistance, the transfer of pattern recognition receptors (PRRs) might confer resistance to current pathogens in crops by recognition of undetected epitopes (Schoonbeek et al., 2015). Hence, expression analysis of selected PRRs, transcription factors, kinase, and R genes was performed on FHB infection in barley to ascertain its role in resistance. Interestingly, silencing of the chitin elicitor-binding protein (CEBiP) orthologous gene in barley increases susceptibility to the fungus *Magnaporthe oryzae* (Tanaka et al., 2010), suggesting that CEBiP may be involved in chitin perception. *TaMPK3*, which encodes a MAP kinase, involved in resistance to *Mycosphaerella graminicola* (Rudd et al., 2008). A wheat pore-forming toxin-like (PFT) homolog of *HvPFT* gene was highly expressed and regulatory FHB-resistant gene in Sumai 3 (He et al., 2018). The transcription factor *HvWRKY23* regulates flavonoid glycoside and hydroxycinnamic acid amide biosynthetic genes in barley to combat FHB (Karre et al., 2019), whereas *TaWRKY70* in wheat *QTL-2DL* regulates downstream metabolite biosynthetic genes to resist *Fusarium graminearum* infection spread within spike (Kage et al., 2017). Expression of salicylate-responsive *pathogenesis-related 1* (*HvPR1*) was strongly induced by *Fusarium graminearum* strain Fg-IFA65GFP in barley leaves (Koch et al., 2016). The homolog ubiquitin ligase gene *PUB23*-like was highly induced in response to *Fusarium* PAMP treatment in *Arabidopsis* (Trujillo et al., 2008). The wheat homologs of *HvLOX2, HvCOI1, HvICS1, HvPAL1, HvNPR1*, and *HvNPR3* were reported to have a regulatory role in FHB resistance (Thapa et al., 2018). The wheat homolog of *HvCamBP* is a particularly good marker due to robust upregulation upon PAMP treatment (Schoonbeek et al., 2015), whereas the CRISPR/Cas9 editing of susceptibility *SWEET1* gene provides the potential for the development of FHB-resistant barley (Ahmad et al., 2020). As most of the genes get upregulated upon FHB infection, their selection in gene for expression analysis may reveal a resistance mechanism and can be used as PTI marker genes in barley.

The research evaluated the ability of six LAB isolates to inhibit *Fusarium* spp. and the *Fusarium* infection of spring barley through *in vitro* and glasshouse experiments. *In vitro* dual-culture assays were followed by detached leaf assays. FHB trials were established under glasshouse conditions, and a gene expression study examined the potential priming capabilities of starter cultures of LAB on the regulation of spring barley defense gene expression.

## Materials and Methods

### Plant Material

The Spring barley cv. “Sanette” used for *in vitro* and glasshouse studies was kindly provided by Gold Crop Ltd. (Cluide, Dunleer, Co., Louth, Ireland).

### Lactic Acid Bacteria, *Fusarium*, and Their Culture

LAB isolates (Table 1) were chosen based on their ability to inhibit the growth of *F. culmorum* strain TMW4.2043 *in vitro* (Axel et al., 2016), which reported that *F. culmorum* strain 126 TMW4.2043 growth was inhibited by these selected LABs depending upon the synthesis of antifungal-active acids such as 3-phenyllactic acid, 4-hydroxyphenyllactic acid and 2-hydroxyisocaproic acid in quantities between 0.1 and 360 mg/kg. *L. reuteri* M13, lacking antifungal activity, was selected as a negative control (Lynch et al., 2016; Peyer et al., 2016). LAB were re-cultured regularly on de Man, Rogosa, and Sharpe (MRS) agar-filled Petri plates (M.R.S AGAR, Oxoid Ltd., Hampshire, United Kingdom) as per Meroth et al. (2003). Cultures were incubated at 30°C (*L. amylovorus* and *L. brevis* strains) and 37°C (*L. reuteri* strains) for 48 h under static conditions, and they were stored short term at 4°C. Single colonies were sub-cultured and used to inoculate 10 ml of de man, Rogosa, and Sharpe broth (MRS broth, Oxoid Ltd., Hampshire, United Kingdom). After a 24-h period, bacteria were sub-cultured at 1% into MRS broth at the respective temperatures for a further 48 h. Growth was observed through the assessment of the optical density of MRS broth at 620 nm (OD620) (Kedia et al., 2007). *Fusarium graminearum* strain GZ3639 and *Fusarium culmorum* strain FCF 200 were obtained from the UCD School of Biology and Environmental Science fungal collection (the former kindly provided by Robert Proctor, USDA). Fresh asexual macroconidia of both *Fusarium* spp. were cultured, and conidia were prepared as per the method outlined by Brennan et al. (2003). Following incubation, cultures were washed, and spores were counted using a haemocytometer (Hycor Biomedical) and adjusted to the 2 × 10^5^ spores/ml using 0.2% Tween 20 (VWR Chemicals, Pennsylvania, United States).

**TABLE 1:** Lactic acid bacteria.

Species	Isolate code	References
*Lactobacillus amylovorus*	FST 2.11	UCC, Cork, Ireland
*Lactobacillus amylovorus*	DSM 20552 (–)	UCC, Cork, Ireland
*Lactobacillus reuteri*	R29 (+)	Axel et al., 2016
*Lactobacillus reuteri*	*M13* (*–)*	Lynch et al., 2016
*Lactobacillus amylovorus*	*DSM20053* (*–)*	UCC, Cork Ireland
*Lactobacillus brevis*	*R2*Δ (+)	Axel et al., 2016

### Fungicide Preparation

The fungicide Fandango^®^ (Bayer CropScience Ltd., Dublin, Ireland) was used as a comparative treatment based on its commercial use against foliar, spikelet, and stem base diseases of small grain cereals. It was prepared as per manufacturers’ recommendations at the rate of 1.25 L/ha (Bayer CropScience Ltd., Dublin, Ireland).

### Dual-Culture Assay

Antifungal activity of the selected LAB against *F. graminearum* strain GZ3639 was observed using a modified version of the dual-culture assay (Khan et al., 2006). Potato dextrose agar (PDA; Oxoid Ltd., United Kingdom) plates were inoculated with mycelial plugs (10 mm diameter) from 7-day-old *Fusarium* cultures placed at the center of each plate. Three sterile 1.0-cm Whatman filter paper disks (Whatman plc, Buckinghamshire, United Kingdom) were equidistantly placed at the perimeter of each plate 26 mm from mycelial plugs. Ten microliter aliquots of LAB (log CFU/ml = 9) were pipetted onto each disk (MRS broth was used as a negative control). Plates were incubated at 21°C ± 1°C in darkness. The antifungal activity was evaluated by measuring the area of the inhibition surrounding individual filter paper disks 168 h post-incubation, using ImageJ software (NIH, United States). A total of three trials were completed. Each trial consisted of three biological replications for each treatment and control.

### Detached Leaf Assay

This experiment was a modification of the detached leaf assay (Chen et al., 2006). Four seeds were sown in 4-cm pots containing John Innes Number 2 compost (John Innes Manufacturer Association, Berkshire, United Kingdom) and grown at 20°C ± 4°C under artificial light conditions of 16-h light/8-h dark cycle at 150 μmol m-2 s-1. The relative humidity was maintained at 50% ± 5%. Seedlings were thinned to two per pot 7 days after germination. After 21–25 days, second true leaves, growth stage 12 (Zadoks et al., 1974), were detached. Leaf sections (6 cm) were placed adaxial side up in square Petri dishes on 10% plant nutrient agar (Duchefa, Netherlands), pH 5.7, comprising 0.67 μM benzimidazole (Oxoid Ltd., Hampshire, United Kingdom). Treatments were positioned centrally along the midrib of each leaf section. They consisted of 10 μl containing 1 × 10^6^ *F. graminearum* strain GZ3639 conidia ml^–1^ 0.02% Tween 20 and 20 μl aliquot of the chosen LAB. The negative control treatment combination was 10 μl of 0.02% (v/v) Tween 20 and 20 μl of MRS broth. The positive fungal control treatment combination was 10 μl of 1 × 10^6^ *F. graminearum* strain GZ3639 conidia ml^–1^ 0.02% Tween 20 and 20 μl of MRS broth. The fungicide Fandango^®^ was incorporated as a comparative treatment. The same inoculation method and pattern were followed for *F. culmorum* experiment wherein *F. culmorum* spores were used. The treated leaves in plates were incubated under the conditions. The number of conidia per leaf section was determined at 5 dpi as per Chen et al. (2006). Starting at 48 hpi, mycelial spread was measured using ImageJ computer software (NIH, United States). Disease severity was assessed per the relative lesion area (RLA) at 48, 72, 96, and 120 h post-inoculation. RLA was calculated using the formula %RLA = 100 × (total area of lesions)/(total leaf area). A total of three replicate trials were completed. Each replicate trial consisted of 12 biological replicates for each treatment and control.

### FHB Severity and Mycotoxin Analysis

An adult plant experiment was conducted to establish the effects of LAB on FHB development and mycotoxin accumulation. Four pre-germinated seedlings were transferred at 10-cm spacings into 3-L containers of John Innes Number 3 compost (John Innes Manufacturer Association, Berkshire, United Kingdom). Plants were placed in an unlit, ventilated greenhouse. Natural light levels were maintained under shading engaged at 60,000 lux m−1 and retracted at 50,000 lux m-1. Seedlings were culled to two per container after 20 days. Plants were irrigated three times weekly along with an added liquid feed of 18–18–18 at a dilution of 1:1,000 weekly with a granular feed of 22-5-6 + 2 Mg + TE (4–5 months, Osmocote^®^ Topdress FT, Everris International BV, Netherlands, containing 22% nitrogen, 2.2% phosphorous, 5% potassium, 1.2% magnesium and trace elements) applied 14 days after transplanting and ca.1 week prior to applications of biocontrol agents.

Spray inoculations (4 ml) of LAB isolates at a concentration of log CFU/ml = 9 (onto secondary tillers) were made ca. 24 h prior to mid-anthesis. Control heads were treated with MRS broth. At mid-anthesis [growth stage 65 (Zadoks et al., 1974)], heads were treated with 4 ml of 0.02% (v/v) Tween 20 (control) or 4 ml of 0.02% (v/v) Tween 20 (mock) supplemented with 2 × 10^5^ conidia ml^–1^ of *F. graminearum* strain GZ3639. Directly after inoculations, all treated heads were enclosed in resealable bags for 3 days. A total of three trials were completed. Each trial consisted of eight biological replicates for each treatment/control arranged in a randomized block design. Disease severity was estimated by counting the number of infected spikelets per ear and expressing this as a percentage of total spikelet infections at 5, 10, and 14 dpi. *Fusarium* infection was identified as brown discolored lesions with premature blanching of individual spikelets (McCallum and Tekauz, 2002; McMullen et al., 2012). The treated spikelets were harvested at plant senescence (growth stage 99 (Zadoks et al., 1974). A total of three biological replicates were completed. Each biological replicate consisted of 32 trials for each treatment and control. Spikelets were dried in a desiccator with silica gel beads for 14 days. Spikelets were triturated using a Glen Creston Hammer Mill and sieved through a 1.0-mm mesh sieve. Sieved samples were bulked according to the treatment per replicate. Mycotoxin testing was carried out for DON, aflatoxin, ZEA T-2 toxin, and ochratoxin by^©^ Sciantec Analytical Laboratories using specific R-Biopharm RIDASCREEN^®^ enzyme-linked immunosorbent assay (ELISA) tests. The absorbance value measured at 450 nm with the RIDA^®^SOFT Win (Art. No. Z9999) software package (R-Biopharm, Darmstadt, Germany) was used to deduce the toxin content of each sample.

### Analysis of Defense Gene Expression in Adult Barley Plants

A separate experiment used quantitative reverse transcriptase-polymerase chain reaction (qRT-PCR) analysis to elucidate the effect of LAB isolates on the regulation of important defense genes and transcription factors in flowering barley heads (Table 2). Spikelets were treated with the same LAB, *Fusarium*, and control treatments detailed above. The treated spikelets were harvested at 12-, 24-, 48-, 72- and 120-h time points post-fungal inoculation. RNA was extracted using Trizol™ reagent according to the manufacturers’ specifications (Thermo Fischer Scientific, Massachusetts, United States) (Chomczynski, 1993). Reverse transcription of total RNA was performed as previously described (Meroth et al., 2003; Walter et al., 2008). Accumulated threshold cycle (Ct) values were obtained by qPCR, and the barley genes *HvActin* and *HvU-61* were used as housekeeping genes to calculate the relative expression of the selected defense genes. The formula described previously (Livak and Schmittgen, 2001) was used to calculate relative gene expression: 2-Δ (Ct target gene—Ct housekeeping gene). This gene expression study was based on an FHB experiment comprised of three biological replicates with 32 trials for each treatment in each biological replicate. Each treatment time point consisted of 3–4 pooled spikelets from two separate plants per replicate. All real-time RT-PCR analyses were performed two times (two cDNAs generated from independent reverse transcriptions from each individual sample of RNA) for all the treatments.

**TABLE 2:** List of genes and primers used for real-time expression studies.

Genes/Annotation	Forward primer (5′-3′)	Reverse primer (5′-3′)	References
*Hvtub/tubulin*α*-2 chain*	GTCCACCCACTCCCTCCTTG	CGGCGGCAGATGTCATAGATG	Ali et al., 2014
*HvActin/actin*	CCACGAGACGACCTACAAC	CACTGAGCACGATGTTTCC	Ferdous et al., 2015
*HvGADPH/glycolytic glyceraldehye-3-phosphate dehydrogenase*	GCCAAGACCCAGTAGAGC	CACATTTATTCCCATAGACAAAGG	
*HvPR1/pathogenesis-related protein*	ACTACTACCTTTCACCCCACAAC	GATCCTCTGGTTGGCGTA	
*HvPFT3B/pore-forming protein-like*	CACGTTCGACACCATCCTG	GATGAACACCGAGTAGCTCC	
*FgActin/Fusarium graminearum*γ-*actin*	ATGGTGTCACTCACGTTGTCC	CAGTGGTGGAGAAGGTGTAACC	Brown et al., 2011
*HcWRKY70/transcription factor*	GACAATCCCTCCACACCAAG	TCACTCCTGCTCCACCTAG	
*HvCAMBP1/calmodulin-binding protein*	CGCGTTCGAGGAGAAACAAG	CGTACCTTGACCAGCCTTGT	
*HvWRKY23/WRKY transcription factor*	GAGCGTAGACGTCAGCACCA	CACGGATGCTAATGGCCACC	
*HvPUB23/ubiquitin ligase protein*	CGTTCATCAGAATGCTCAGCTG	TTCTCTTTTGTAGGCACGAACCA	
*HvLOX2/lipoxygenase for jasmonic acid synthesis*	GCACCGCCTGCTGCACCCGC	CGGCTGACGAGGTCCTCCGGCG	
*HvCOI1/coronatine-insensitive protein 1*	CATTGTGCGAGTGAACTGTGACA	CGCGGAAACCAGACAAGCT	
*HvICS1/isochorismate synthase 1*	CGGACGGCCCCGCCGAGGAC	CGCGGCGGTCGACGCGGCGGGA	
*HvPAL1/phenylalanine ammonia lyase 1*	CGACGAGGTCAAGCGCATGGT	CGGCTGCTCTCCTTGACGCGG	Dempsey et al., 2011
*HvNPR1/regulatory receptor protein 1*	CGCGGACGTGGAGGCGCTCCGC	CCGGTTGCCCTCGGCGCCGCCG	
*HvNPR3/regulatory receptor protein3*	ATGGAGCCGTCGTCGTCCATCA	TCCGCCACGTCGACGTCGGCGT	Zhang et al., 2006
*HvMPK3/MAP kinase 3*	ACCCTTACCTAGAGCGGC TTC	ACTCCAGGGCTTCGTTGAATA	

### Statistical Analysis

All data from *in vitro* and glasshouse experiments were analyzed in R v4.0.2 (R Core Team). Normality was tested using the Shapiro–Wilk test. Non-normal data were analyzed by a pairwise Kruskal–Wallis test. Correlations of data were tested using Spearman’s rank correlation coefficient analysis. Data from gene expression studies were analyzed using GraphPad Prism6^®^ (GraphPad Software, San Diego, California, United States).

## Results

### Assessment of the Inhibitory Effect of Lactic Acid Bacteria Against *Fusarium graminearum* in Dual Cultures

**FIGURE 1:**
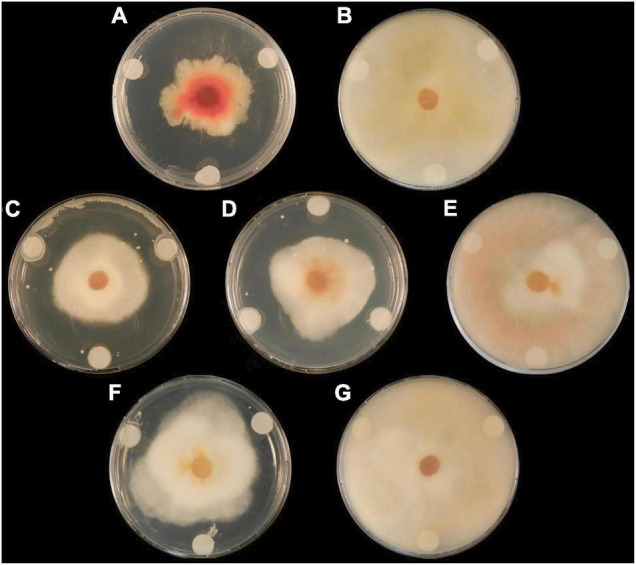
The effect of lactic acid bacteria on radial growth of *F. graminearum* GZ3639 on potato dextrose agar plates at 7 days. Treatments **(A)** (*L. amylovorus* FST 2.11), **(C)** (*L. brevis* R2Δ), and **(D)** (*L. amylovorus* DSM 20053) displayed clear zones of fungal inhibition compared with the control (*P* < 0.05), whereas treatments **(B)** (*L. reuteri* M13), **(E)** (*L. reuteri* R29), and **(F)** (*L. amylovorus* DSM 20552) displayed lowered antifungal activity (*P* > 0.05). Control treatment **(G)** (MRS broth) displayed no inhibition.

Dual-culture assays assessed the capacity of six *Lactobacillus* strains to inhibit the spread of mycelium from *Fusarium graminearum* GZ3639. The maximum inhibition was provided by *L. brevis* R2Δ with an average reduction of 17% in mycelial growth (*p* < 0.05) (Table 3). Both *L. amylovorus* FST 2.11 and *L. amylovorus* DSM 20053 significantly reduced mycelial spread compared with the control (*P* < 0.05). The three remaining *Lactobacillus* isolates, namely, *L. amylovorus DSM 20552, L. reuteri M13* and *L. reuteri* R29, failed to significantly inhibit mycelial spread compared with the control (*p* > 0.05). No clear zone of inhibition was observed at the point of contact between the control (MRS broth) and the fungus. In addition to mycelial impedance, it was observed that *L. amylovorus* FST 2.11 altered hyphal color from cream/light pink to a bright pink/red hue (Figure 1).

**TABLE 3:** Percentage of growth inhibition of *F. graminearum* by six *Lactobacillus species* under *in vitro* dual-culture conditions.

Bacterial *spp.*	Isolate	Average area of fungal inhibition (mm^2^)	% Inhibition of fungal mycelium (after 7 days)
*L. brevis*	R2Δ	485.2425	17.16 *
*L. amylovorus*	DSM 20053	255.42	9.03 *
*L. amylovorus*	FST 2.11/DSM19280	296.6725	10.49 *
*L. amylovorus*	DSM 20552	101.5875	3.59
*L. reuteri*	R29	58.46	2.06
*L. reuteri*	M13	142.7625	5.04
MRS broth		4.7525	0.16

*Indicates a significant difference in the% inhibition of fungal mycelium compared with the control treatment (P < 0.05).

### Assessment of the Ability of Lactic Acid Bacteria to Inhibit *Fusarium* Infection, as Determined Using a Detached Leaf Assay

**FIGURE 2:**
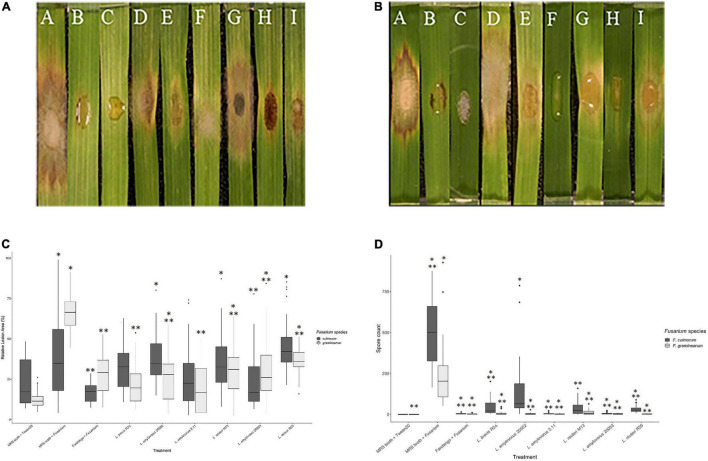
Effect of lactic acid bacteria on the mycelial growth of *Fusarium spp.* using detached leaf assay. **(A)** Treatments were applied simultaneously. Disease estimation was calculated by measuring the relative lesion area at 120 h post-inoculation. * Indicates treatments significantly different than the control MRS broth + Tween 20 (*F. graminearum*). ** Indicates treatments significantly different than the control MRS broth + *F. graminearum*. **(B)** Symptomatic diseased lesions on barley leaves. The barley leaves were treated with *F. graminearum* and controls [(A) MRS broth + *F. graminearum*, (B) Fandango^®^ + *F. graminearum* (C) MRS broth + Tween 20]. MRS broth and lactic acid bacteria [(D) *L. reuteri* R29, (E) *L. brevis* R2Δ, (F) *L. amylovorus* FST2.11, (G) *L. reuteri* M13, (H) *L. amylovorus* DSM 20053, and (I) *L. amylovorus* DSM 20552]. The effects of the treatments above were observed 120 h post-inoculation. **(C)** Symptomatic diseased lesions on barley leaves treated with *F. culmorum* and controls [(A) MRS broth + *F. culmorum*, (B) Fandango^®^ + *F. culmorum* (C) MRS broth + Tween 20]. MRS broth and lactic acid bacteria [(D) *L. reuteri* R29, (E) *L. brevis* 2Δ, (F) *L. amylovorus* FST 2.11, (G) *L. reuteri* M13, (H) *L. amylovorus* DSM 20053, and (I) *L. amylovorus* DSM 20552]. The effects of treatments above observed 120 h post-inoculation. **(D)** An assessment of LAB treatments on sporulation of *Fusarium spp.* by detached leaf assay. Conidia production was determined 5 days post-inoculation. *** Indicates treatments significantly different than the control MRS broth + Tween 20 (*F. culmorum*). **** Indicates treatments significantly different than the control MRS broth + *F. culmorum*. * Indicates treatments significantly different than the control MRS broth + Tween 20 (*F. graminearum*). ** Indicates treatments significantly different than the control MRS broth + *F. graminearum.*

Based on the mycelial growth inhibition *in vitro* assay, the selected LAB was assessed *in plant* for the fungal spread and sporulation suppression of *F. graminearum* and *F. culmorum* using a modified leaf detachment assay (Chen et al., 2006). No *Fusarium*-like lesions appeared on control treatments (MRS broth and Tween 20). On all other treatments, at 120 hours post-inoculation (hpi), all leaves displayed brown necrotic lesions typical of *Fusarium* infection (Koch et al., 2013). Lesion area on leaves treated with the positive control of both test fungi was significantly larger than that of the negative control at 120 hpi, although these differences were only significant from 72 hpi onward (*p* < 0.001). The positive control across both *Fusarium* spp. resulted in the highest average relative lesion area, as compared to all other treatments (Figure 2A). Lesion size was expressed as a percentage of infection to control treatment 10 μl of 0.02% (v/v) Tween 20 and 20 μl of MRS broth. For the leaves treated with LAB in combination with *F. culmorum*, the most effective isolates minimizing lesion size were *L. amylovorus* DSM 20053 (24%), *L. amylovorus* FST 2.11 (26%) and *L. brevis* R2Δ (32%).

The fungicide Fandango^®^ provided significantly higher levels of disease suppression compared with all LAB isolates (*P* < 0.001) except *L. amylovorus* DSM 20552. Against *F. graminearum*, all LAB isolates significantly reduced diseased lesion area compared with the positive control (MRS + *F. graminearum*) (*P* < 0.05). The effect of LAB on sporulation of both *Fusarium* species was examined. Leaves treated with the positive control resulted in significantly greater spore quantities than those of the negative control (*P* < 0.001). All LAB isolates displayed an ability to reduce sporulation of *F. graminearum* compared with the positive control (*P* < 0.001) (Figure 2D). Leaves treated with Fandango^®^ + *Fusarium graminearum* resulted in a larger lesioned area than that of the negative control of MRS + Tween 20 (*p* > 0.05). Leaves inoculated with the test fungus *F. graminearum* and MRS broth brought about the largest relative lesion area (68%), while *L. brevis* R2Δ and *L. amylovorus* DSM 20552 were the LAB that caused the greatest lesion inhibition at 24 and 23%, respectively (Figure 2C). Compared with the positive control, sporulation of *F. graminearum* saw the biggest reduction by *L. amylovorus* FST 2.11 with an average spore count of 2.3 per detached leaf (*p* < 0.001). Subsequent bacteria which significantly reduced sporulation of *F. graminearum* included *L. reuteri* R29 and *L. amylovorus* DSM 20053 with average spore counts of 2.61 and 3.00 per detached leaf, respectively (*p* < 0.001).

Comparable results were observed for detached leaves co-inoculated with *F. culmorum and* LAB (Figures 2B,D). The positive treatment resulted in the highest levels of spore production which were significantly higher (504.22) than most LAB treatments (*P* < 0.001). The exception was *L. amylovorus* DSM 20552 which did not have a significantly reduced spore count (*p* > 0.05). LAB isolates with the greatest inhibition of spore count included *L. amylovorus* FST 2.11 and *L. amylovorus* DSM 20053 which did not differ significantly when compared with the negative control (*p* > 0.05). No correlations between treatments were found for LAB co-inoculations against both test fungi.

### Evaluation of the Effect of Lactic Acid Bacteria Treatments on FHB Development and Mycotoxin Accumulation Under Greenhouse Conditions

**FIGURE 3:**
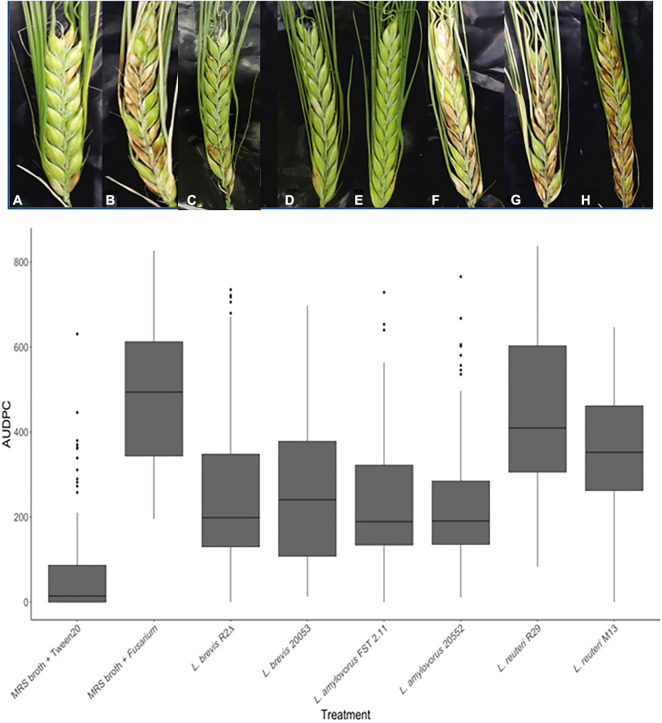
FHB symptoms of spring barley cultivar “Sanette” assessed following LAB and control treatments at 14 days post-inoculation. Chosen spikelets received LAB [(C) (*L. amylovorus* DSM 20552)], [(D) (*L. amylovorus* DSM 20053)], [(E) (*L. brevis* R2Δ)], [(F) (*L. amylovorus* FST 2.11)], [(G) (*L. reuteri* M13)], [(H) (*L. reuteri* R29)] or MRS broth (A,B). Conidia of *F. graminearum* (B) or Tween 20 (mock) (A) were spray applied at mid-anthesis. Disease severity means were analyzed using the Kruskal–Wallis H test. * Indicates treatments significantly different from the control MRS broth + Tween 20. ** Indicates treatments significantly different than the control MRS broth + *F. graminearum*/*F. culmorum.*

An FHB experiment assessed the impact of *L. amylovorus* FST 2.11, *L. amylovorus* DSM 20552, *L. amylovorus* DSM 20053, *L. reuteri* M13, *L. brevis* R2Δ, and *L. reuteri* R29 (Table 1) on disease development and mycotoxin accumulation in grain. Disease severity scoring was assessed at 5, 10, and 14 dai and used to calculate the area under disease pressure curve (AUDPC) (Jeger and Viljanen-Rollinson, 2001; Legzdina and Buerstmayr, 2004; Legzdina et al., 2013). Disease symptoms were minimal but not completely absent on spikelets treated with Tween 20 (mock). This is likely due to the heightened levels of background infection after unnatural levels of inoculum were applied to the neighboring plants. The majority of LAB treatments significantly reduced FHB severity compared with the positive control of MRS + *F. graminearum* (Figure 3). The exception was heads treated with *L. reuteri* R29, which showed no significant reduction compared with the positive control (***P*** > 0.05). A noteworthy observation was the deleterious effect that early inoculations of MRS broth, and MRS broth containing bacterial cultures, had on spike maturation. In some cases, heads treated more than 24 h before mid-anthesis commenced premature cessation.

**FIGURE 4:**
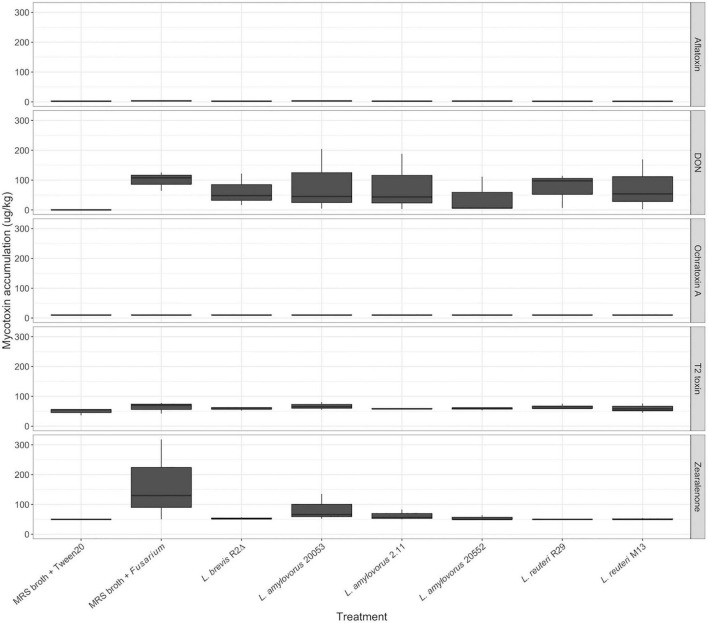
Mycotoxin accumulation in pooled FHB grain samples. Mycotoxin means were separated using the Kruskal–Wallis H test.

Spikelets were analyzed for the mycotoxins DON, aflatoxin, ochratoxin A, T-2 toxin, and zearalenone (Figure 4). Correlation of mycotoxin accumulation was moderate to strong between the three trials (*r* > 0.55) across all treatments. DON was the highest recorded mycotoxin at 204.4 μg/kg. Ochratoxin A levels were undetectable using ELISA. Mycotoxin accumulation of DON, T-2 toxin, ZEA, and aflatoxin was not significantly different across treatments from three replications (*p* > 0.05). Although mycotoxin accumulation was generally higher in the samples treated with MRS broth and *Fusarium* than in samples treated with LAB and *Fusarium*, no significant differences were found in mycotoxin accumulation between LAB treatments, once *P*-values were adjusted for multiple comparison testing.

### The Effect of Lactic Acid Bacteria on Defense Gene Expression in Spikelet Tissue

**FIGURE 5:**
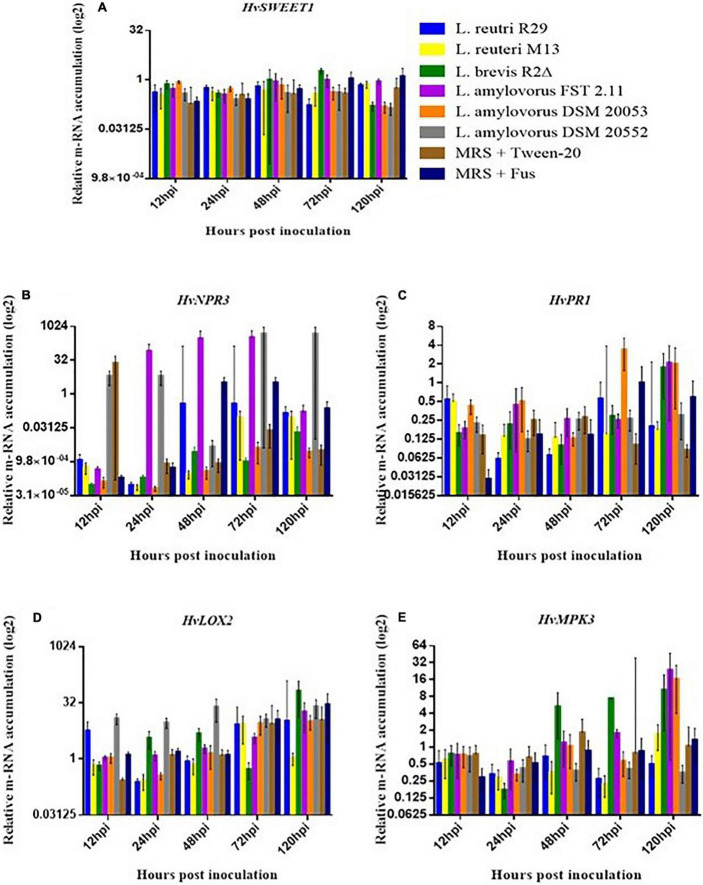
Relative gene expression level in barley head tissue harvested at 12, 24, 48, 72, and 120 hpi of fungal inoculation. **(A)** *HvSEEWT1*, **(B)** *HvNPR3*, **(C)** *HvPR1*, **(D)** *HvLOX2* and **(E)** *HvMPK3*. Error bars indicate ± SEM (*n* = 8). Asterisks show significant differences between expression levels (*P, 0.05).

**FIGURE 6:**
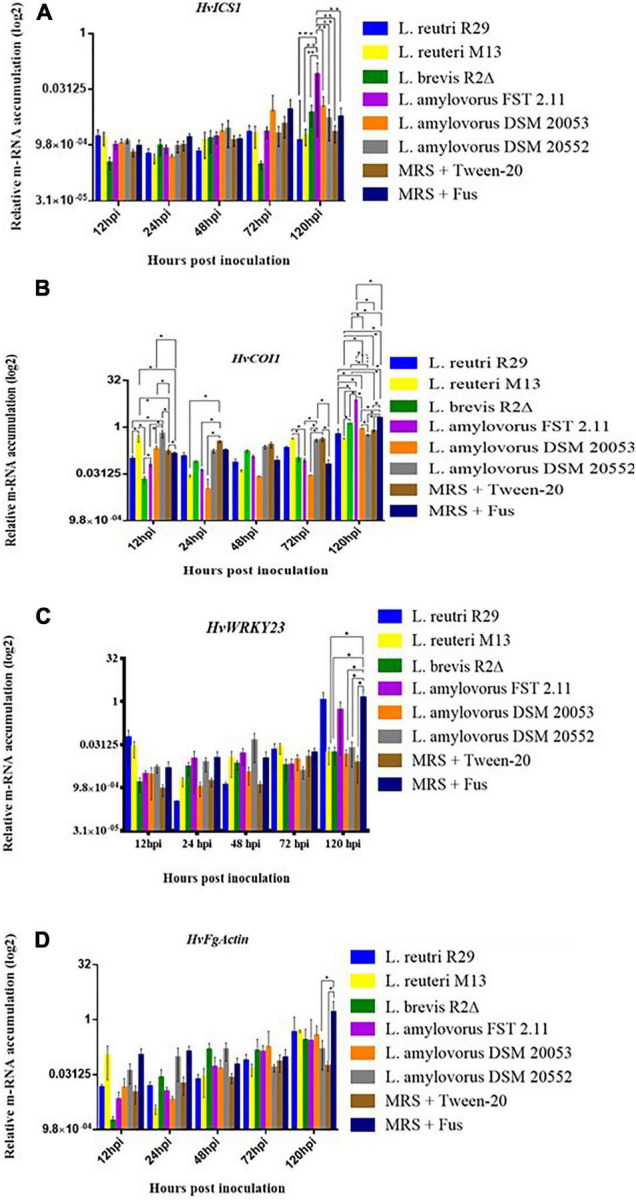
Relative gene expression level in barley head tissue harvested at 12, 24, 48, 72, and 120 hpi of fungal inoculation. **(A)** *HvICS1*, **(B)** *HvCOI1*, **(C)** *HvWRKY23* and **(D)** *FgActin*. Error bars indicate ± SEM (*n* = 8). Asterisks show significant differences between expression levels (*P, 0.05).

Quantitative real-time PCR (qRT-PCR) was used to quantitatively determine the effect of LAB isolates on the expression of 11 important defense-related genes (Table 2). All genes were expressed in barley spikelets treated with 0.2% Tween 20 and MRS broth (mock). The target gene expression was quantified relative to that of the barley housekeeping genes *HvActin* and *HvU-61*. Responsiveness of defense-related genes following LAB and *Fusarium* applications showed that different treatments triggered variable expression levels across the time points. One notable trend was the increased quantity of *HvPUB23, HvICS1, HvWRKY23, HvActin, HvCAMBP1*, and *HvCOI1* transcripts from 72 to 120 hpi. Spray inoculations of spikelets with LAB and *F. graminearum* resulted in no significant changes in the expression of *HvSWEET1, HvNPR3, HvPR1, HvLOX2*, and *HvMPK3* compared with those treated with the positive or negative controls (Figure 5). Levels of mRNA accumulation of *HvICS1* were found to progressively increase from 12 hpi to 120 hpi (Figure 6A). Significant differences in *HvICS1* expression levels were not noted until 120 hpi where *L. amylovorus* FST 2.11-treated spikelets had significantly more transcripts as compared to all other LAB treatments and controls (*p* < 0.05). Significant changes triggered by LAB treatments on the expression of *HvCOI1* were noted from 12 hpi (*p* < 0.05) (Figure 6B). At 120 hpi, all LAB treatments brought about significant changes in levels of *HvCOI1* expression compared with the positive control of MRS broth + *F. graminearum*. Of the six LAB isolates, only *L. amylovorus* FST 2.11 expressed higher levels of *HvCOI1* expression (*P* < 0.05), the remainder expressing lower levels compared with the positive control at 120 hpi (*p* < 0.05). Three treatments of *L. amylovorus* FST 2.11, *L. amylovorus* DSM 20053, and *L. brevis* R2Δ did induce *HvCOI1* expression compared with the negative control of MRS broth + Tween 20 (*p* < 0.05), but only *L. amylovorus* FST 2.11 expressed higher levels of *HvCOI1* compared with the positive control treatment (*p* < 0.05). The remaining LAB treatments all provided lower levels of *HvCOI1* expression compared with the negative control, with *L. reuteri* M13 significantly lower (*p* ≤ 0.006).

The expression of transcription factor *HvWRKY23* from 12 to 120 hpi was inconsistent with induction and downregulation (Figure 6C). From 12 to 72 hpi, no significant effect of treatments was observed. At 120 hpi, a significant reduction in expression levels was noted when the control treatment MRS + *F. graminearum* was compared with LAB treatments of *L. brevis* R2Δ, *L. amylovorus* DSM20053 and *L. amylovorus* DSM20552 (*P* < 0.05). At the same time point, expression levels of *HvWRKY23* were also significantly greater for spikelets treated with MRS broth + *F. graminearum* compared with the mock treatment of MRS broth + Tween 20 (*P* ≤ 0.01). The expression levels of *HvPUB23* were examined with significant differences only observed at 120 hpi. Accumulation of mRNA was significantly greater in *L. reuteri* R29 when compared with *L. reuteri* M13 (*P* ≤ 0.014), *L. amylovorus* DSM 20053 (*P* ≤ 0.022), *L. amylovorus* DSM 2055 (*P* ≤ 0.013), MRS broth + *F. graminearum* (*P* ≤ 0.046) and MRS broth + Tween 20 (*P* ≤ 0.017). Differences in expression levels of *L. reuteri* R29, *L. brevis R2*Δ, and *L. amylovorus* FST 2.11 were not significant (*P* > 0.05). The relative gene expression of *FgActin*, monitored as an indicator of fungal activity, revealed a general incline from 12 to 120 hpi (Figure 6D). LAB treatments of *L. reuteri* M13 and *L. reuteri* R29 provided the highest levels of *FgActin* although these differences were not significant (*p* > 0.05). Two treatments, namely, *L. amylovorus* DSM 20552 and the mock, were shown to significantly reduce levels of *FgActin* mRNA accumulation compared with that of MRS broth + *F. graminearum*-treated heads (*p* < 0.05).

## Discussion

Management of fungal diseases caused by several *Fusarium* spp. including *F. graminearum* has remained a ceaseless challenge since the realization of their impact on yield and quality of grain. Indeed, the incessant threat of cool damp weather, particularly at flowering, and increased concern regarding fungicide use to combat the pathogens that cause head blight, foot rot, and seedling blight mean we must reconsider our options (Arendrup et al., 2013; Shi et al., 2020). In addition to yield losses, it is the propensity of many *Fusarium* spp. to contaminate grain with mycotoxins that impacts the health of humans and animals who consume the affected grain which causes much consternation (Koch et al., 2013; Gallo et al., 2015; Tibola et al., 2016). Reforming our approach to these challenges by providing both economically and environmentally driven alternatives to existing fungicide solutions will allow for sustainably driven tillage systems. Hence, the use of LAB as BCAs to protect crops becomes imminent, as BCAs are cheaper, are environment friendly, leave no toxic residue, and are easy to handle, apply, manufacture and multiply in the soil (Sharma et al., 2013; Mittholiya et al., 2020). There has been a report of *in vivo* efficacy of LAB as BCAs against *Fusarium oxysporum* protection in tomato when used as seed treatment (Hamed et al., 2011). *Lactobacillus coryniformis* subsp. *coryniformis* strain Si3 was also reported to produce PLA, cyclo(Phe-Pro), cyclo(Phe-4-OH-Pro) *in vitro* inhibiting *Fusarium graminearum, Fusarium culmorum, Fusarium sporotrichioides* and *Rhodotorula mucilaginosa* (Magnusson and Schnürer, 2001). Furthermore, the LAB were found to live in positive dynamic and assembly as epiphyte and endophyte with *Origanum vulgare* L (Pontonio et al., 2018), which open a door for natural BCAs. Hence, herein the research project to investigate the potential of LAB to be used as BCAs to control FHB in barley was taken up.

Dual-culture assays revealed that three of the tested LABs, namely, *L. amylovorus* FST 2.11, *L. amylovorus* DSM 20053, and *L. brevis* R2Δ, can reduce the mycelial growth of the test fungus. For the majority, these results agree with the antifungal potential of the selected isolates as shown in previous studies (Lynch et al., 2016; Peyer et al., 2016) with the exception of *L. reuteri* R29 with a diminished antifungal activity. This may be explained by the specificity of *L. reuteri* R29 to *F. culmorum* strain TMW4.2043 (Axel et al., 2016); therefore, the antifungal activity of this LAB isolate could be fungi-specific. In addition, it is acknowledged that growth media and their components used in dual-culture assays may alter the antifungal activity of the test organism (Fiddaman and Rossall, 1994; Wang and Liu, 2008). The result of this assay carried out on a PDA medium can be further validated by carrying out the same assay on nutrient-limited media. These Petri dish assays did not examine the specific manner of antifungal activity, although previous work has noted the production of carboxylic acids by *L. brevis* R2Δ as the basis for the observed antifungal activity (Axel et al., 2016). These experiments agree with the study where *L. reuteri* M13 provided low to absent levels of fungal inhibition (Lynch et al., 2016). A possible reason for mycelium inhibition by the LAB examined could include the production of secondary metabolites which may directly inhibit mycelium spread as seen in other fungal pathogens (Allioui et al., 2016). Other explanations for mycelium inhibition could be the alteration of the growth media such as a change in pH and thus reducing its affinity to support mycelial growth. Alternatively, the LAB could potentially play a hyperparasitic role in the presence of *Fusarium*. Direct competition for space may also have led to a reduction in Fusarium growth needed to acquire nutrients from the media.

A modified detached leaf assay was used as an auxiliary study to quantify the antifungal potential of the selected agents while eliminating the effect of the growth media used in the dual-culture assay. The three aforesaid LAB which provided the most effective control of mycelial spread on dual culture presented similarly in the detached leaf assay. The reduction in differences in the antifungal activity between the best (*L. brevis* R2Δ) and worst (*L. reuteri* R29) performing isolate could be due to the removal of an artificial growth medium. The difference in the induction of host differences in leaves not seen in dual-culture assay may be due to the variation in the inherent potential of LABs to produce antifungal substances, such as cyclic dipeptides, proteinaceous compounds, organic acids, fatty acids, and reuterin (Crowley et al., 2013; Gajbhiye and Kapadnis, 2016). The biocontrol mechanisms of LAB can vary with strains *in vitro* but may have different performance in leaf surfaces. This means that all strains of LABs do not have the same potential to circumvent fungal spoilage owing to different capabilities to produce antifungal metabolites (Lačanin et al., 2017; Russo et al., 2017; Sadiq et al., 2019). The biocontrol mechanism of LAB strains is not limited to the antifungal activity, and it can extend up to interaction with fungal mycotoxins, resulting in inactivation or removal through cell wall binding (Blagojev et al., 2012; Ahlberg et al., 2015; Juodeikiene et al., 2018). The *in vitro* inhibitory mechanism of LAB strains PM411 and TC92 against *Pseudomonas syringae* pv. actinidiae in kiwifruit, *Xanthomonas arboricola* pv. pruni in Prunus and *Xanthomonas fragariae* in strawberry was reported to be pH-lowering effect and the production of lactic acid rather than the production of antifungal activity. However, both strains showed similar survival rates on leaf surfaces (Daranas Boadella et al., 2019). There are reports of LAB having antifungal activity but with variation. Recently, 10 different genotypes of *Lactobacillus plantarum* confirmed having antifungal activity, but all of them were genetically heterogeneous (Dong et al., 2017). In addition, the presence of naturally occurring saprophytes on detached leaves could have a synergistic effect when co-inoculated with the chosen LAB. Although these experiments did not examine the exact mode of inhibition, as a proxy to further trials they give ample evidence to show potential for *in planta* investigations. To allow for a clearer understanding of biocontrol agents, test fungus and plant, biofilm investigations along with whole leaf clearing and live–dead staining techniques could be explored (Bruzzese and Hasan, 1983; Bais et al., 2004).

Though there are more *in vitro* studies examining the potential of biocontrol agents *in vivo*, there is a need to find biocontrol agents that provide control at levels similar to or exceeding that of commercially available fungicides, and glasshouse studies were established (Palazzini et al., 2007; Matarese et al., 2012). To date, few studies have examined the relationship between LAB-mediated protection against FHB and mycotoxin accumulation (Oliveira et al., 2014; Baffoni et al., 2015; Zhao et al., 2017, 2019). In this study, LAB isolates of *L. amylovorus* DSM 20552, *L. amylovorus* DSM 20053, and *L. brevis* R2Δ provided evidence to be most suited as spray inoculations for the control of FHB *in planta* and reduction in the levels of mycotoxins on an individual basis. The main reason for these LAB isolates providing FHB resistance is the reduction in DON accumulation, the main virulence factor for *Fusarium*, inhibiting disease spread within barley heads (Sella et al., 2014; Gunupuru et al., 2017). Synergy studies have shown that when combined, LAB compounds have a higher relative antifungal activity (Niku-Paavola et al., 1999; Ndagano et al., 2011). Future glasshouse studies applying combinations of these isolates would offer more insight as to the potentially deleterious or beneficial effects co-inoculations might have on FHB control and mycotoxin moderation.

Previous studies have clearly shown that biocontrol agents alter plant defense systems upon host–agent contact (Khan et al., 2006; Palazzini et al., 2018; Köhl et al., 2019). The rough exploration of the changes in defense pathways was taken up in this study, as it may prove to be beneficial for rapid *in vitro* selection of future biocontrol agents (Conn et al., 2008). The gene expression of *FgActin* showed the increase in mRNA level of *Fusarium* indicating FHB development with time. But interestingly, *L. amylovorus* DSM 20552 and the mock were shown to significantly reduce levels of *FgActin* mRNA accumulation compared with that of MRS broth + *F. graminearum*-treated heads, suggesting the inhibition of FHB development. The *HvICS1* transcript level increment was found progressively from 12 hpi to 120 hpi. This can be related to FHB development with time, and the increase in *HvICS1* seems logical as *HvICS1* is the important gene for SA (salicylic acid) synthesis and SA signaling has been proven to confer FHB resistance (Makandar et al., 2012; Hao et al., 2018; Thapa et al., 2018). This may suggest that *HvICS1* plays a role in SA accumulation upon FHB infection in barley, which, in turn, confers a basal resistance to *F. graminearum* by modulating the accumulation of H_2_O_2_, O^–2^ and reactive oxygen-associated enzymatic activities (Antalová et al., 2020). The significant changes triggered by LAB treatments on the expression of *HvCOI1* at 12 hpi (*p* < 0.05) do suggest toward the role of LAB in priming barley heads against FHB with time. This is because coronatine-insensitive protein 1(COI1) is a reported receptor of jasmonic acid (JA) signaling and has been known to play a crucial role in FHB resistance in plants (Makandar et al., 2010; Kazan and Gardiner, 2018; Srivastava et al., 2018).

To conclude, this study has explored the effectiveness of *Lactobacillus* isolates as biocontrol agents *in vitro* and under glasshouse conditions. Several isolates provide promising evidence with *L. brevis* R2Δ, *L. amylovorus* DSM 20552, and *L. amylovorus* FST 2.11 showing the most promise regarding mycelial spread *in vitro*, disease severity, and mycotoxin accumulation. Gene expression results add to the burgeoning library recording the underlying interactions between LAB biocontrol agents and their host, resulting in FHB resistance. This work provides a step forward in developing new biological approaches to combat important fungal pathogens during a time when available chemical controls are declining. These results show that LAB control of fungal pathogens is species-specific and caution must be noted with regard to variance in biocontrol properties if the pathogen changes. These LAB can be exploited for their antifungal activity and can be considered useful to control the pathogens and crop improvement. In summary, the results illustrate the potential for a multi-pronged approach to control FHB through direct fungal inhibition, toxin reduction, and host defense pathway induction. This provides an alternative stronghold leading to sustainable agriculture, green control of pathogens, food security, environment friendly, and circular bioeconomy.

## Data Availability Statement

The original contributions presented in this study are included in the article/Supplementary Material, further inquiries can be directed to the corresponding author.

## Author Contributions

JB obtained funding for the project. MB, GT, and JB designed all the experiments. JB and GT provided guidance during the experiment implementation. MB and GT carried out all the experiments. FD and JB reviewed the final manuscript. All authors contributed to the article and approved the submitted version.

## Conflict of Interest

The authors declare that the research was conducted in the absence of any commercial or financial relationships that could be construed as a potential conflict of interest.

## Publisher’s Note

All claims expressed in this article are solely those of the authors and do not necessarily represent those of their affiliated organizations, or those of the publisher, the editors and the reviewers. Any product that may be evaluated in this article, or claim that may be made by its manufacturer, is not guaranteed or endorsed by the publisher.
