# Supplementary material for: Lactic Acid Bacteria as Potential Biocontrol Agents for Fusarium Head Blight Disease of Spring Barley
Source: Front Microbiol. 2022 Jul 22;13:912632. doi: 10.3389/fmicb.2022.912632 (PMC9355582; doi:10.3389/fmicb.2022.912632)
Supplement: Supplementary file 1 [file Data_Sheet_1.PDF]

Explore

[DataSet0]

Case Processing Summary

	Cases					
	Valid		Missing		Total	
	N	Percent	N	Percent	N	Percent
µg/kg	82	68.3%	38	31.7%	120	100.0%

Descriptives

			Statistic	Std. Error
µg/kg	Mean		50.2500	6.08252
	95% Confidence Interval for Mean	Lower Bound	38.1477	
		Upper Bound	62.3523	
	5% Trimmed Mean		43.6668	
	Median		52.0000	
	Variance		3033.757	
	Std. Deviation		55.07955	
	Minimum		.20	
	Maximum		318.00	
	Range		317.80	
	Interquartile Range		61.30	
	Skewness		2.016	.266
	Kurtosis		6.464	.526

Tests of Normality

	Kolmogorov-Smirnov[a]			Shapiro-Wilk		
	Statistic	df	Sig.	Statistic	df	Sig.
µg/kg	.182	82	.000	.789	82	.000

a. Lilliefors Significance Correction

µg/kg

```
µg/kg Stem-and-Leaf Plot

 Frequency    Stem &  Leaf

    31.00        0 .  0011122222222222223333333344466
     2.00        1 .  06
      .00        2 .
     1.00        3 .  6
     5.00        4 .  23558
    15.00        5 .  222334556778889
    10.00        6 .  0111244456
```

```
    4.00        7 .  0568
    2.00        8 .  13
    1.00        9 .  7
    1.00       10 .  7
    2.00       11 .  14
    2.00       12 .  15
    2.00       13 .  05
    4.00 Extremes    (>=169)

Stem width:     10.00
Each leaf:        1 case(s)
```

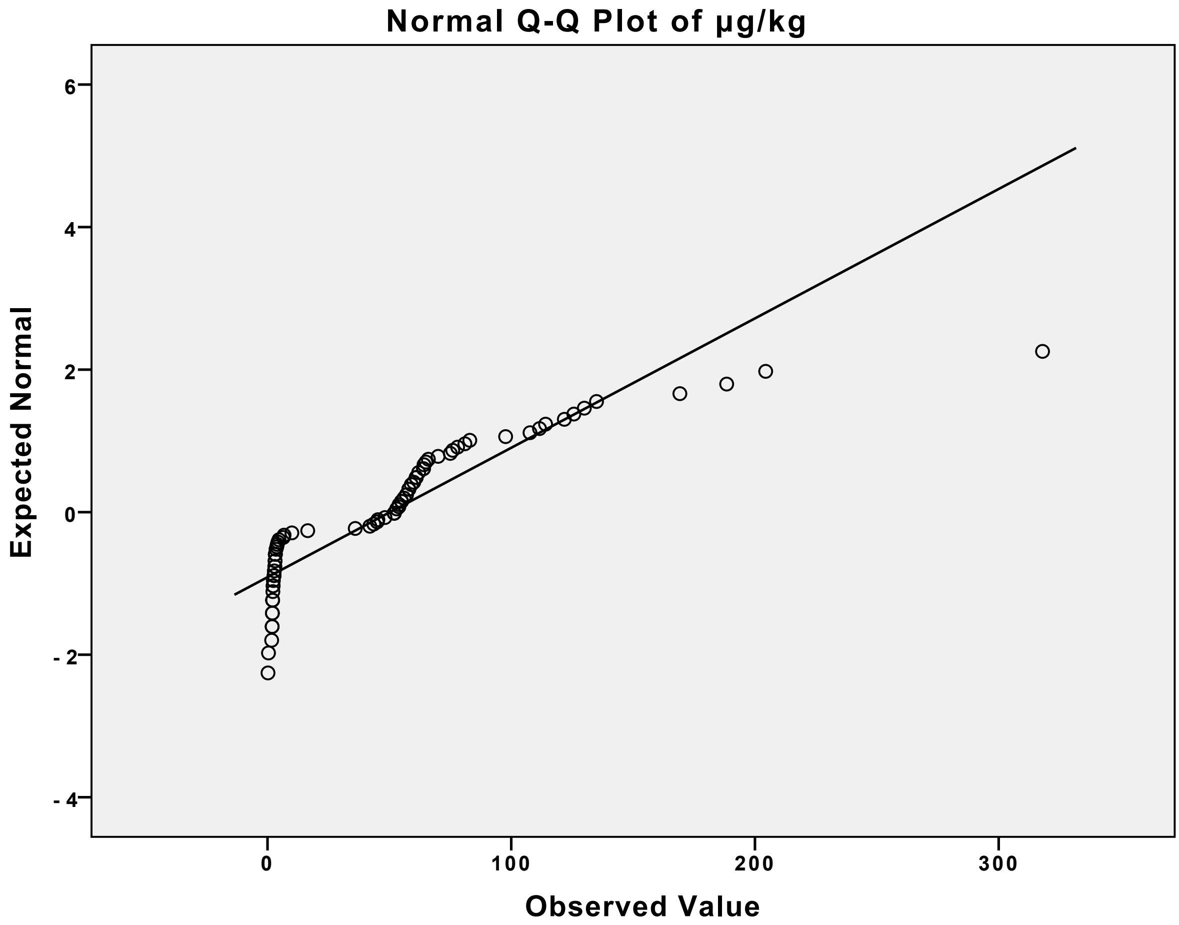

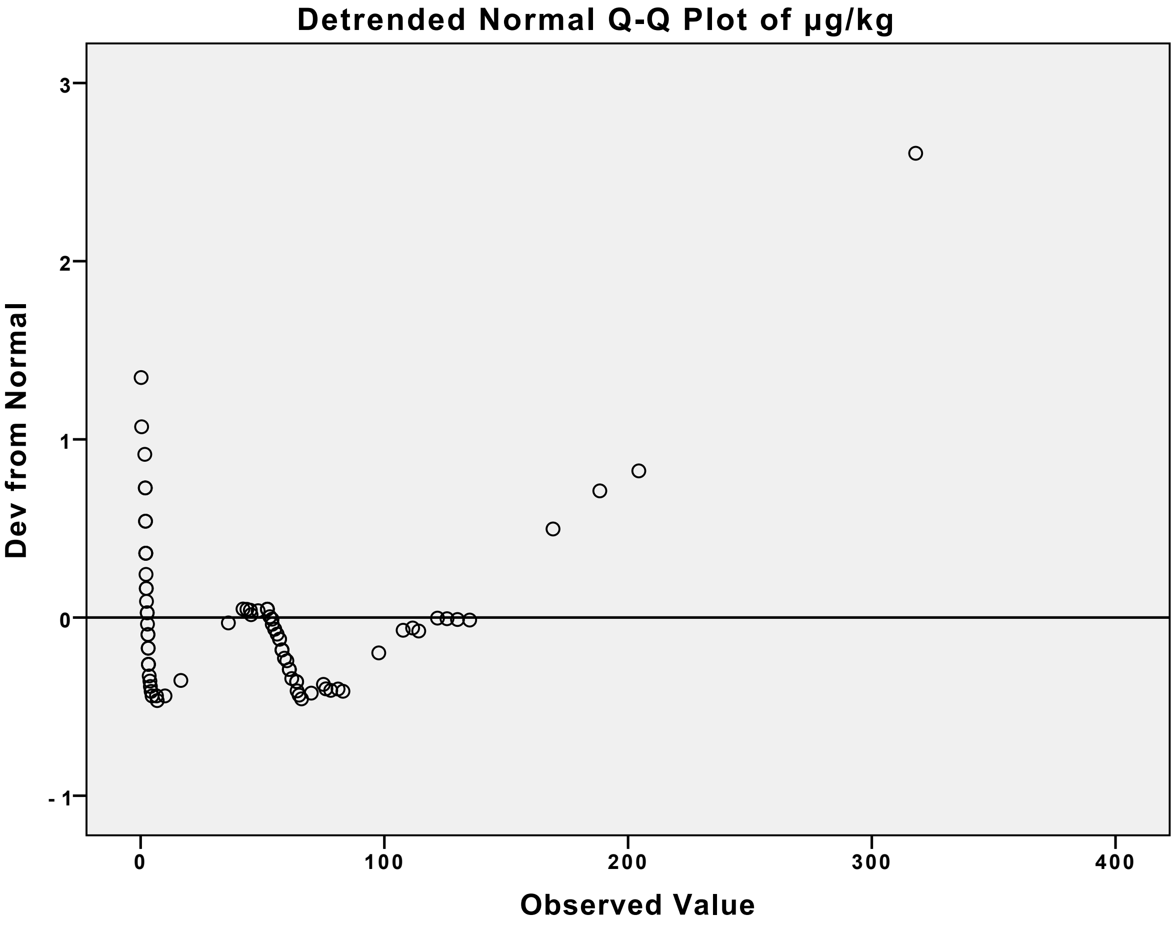

Detrended Normal Q-Q Plot of µg/kg
Dev from Normal
3
2
1
0
- 1
0
100
200
300
400
Observed Value

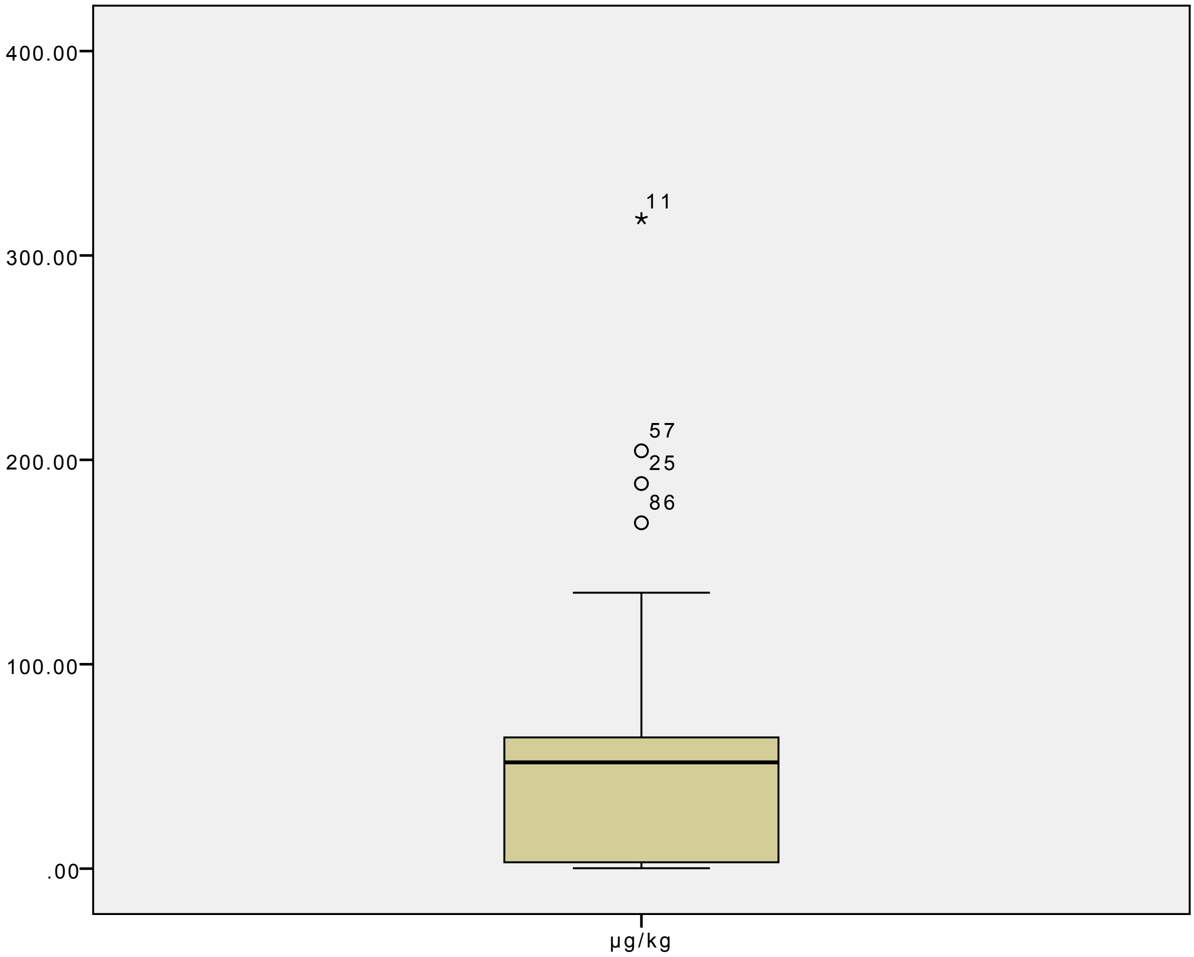

Nonparametric Tests

Mycotoxin = Aflatoxin

Hypothesis Test Summary

	Null Hypothesis	Test	Sig.	Decision
1	The distribution of µg/kg is the same across categories of Treatment.	Independent-Samples Kruskal-Wallis Test	.762	Retain the null hypothesis.

Asymptotic significances are displayed. The significance level is .05.

Mycotoxin = DON

Hypothesis Test Summary

	Null Hypothesis	Test	Sig.	Decision
1	The distribution of μg/kg is the same across categories of Treatment.	Independent-Samples Kruskal-Wallis Test	.430	Retain the null hypothesis.

Asymptotic significances are displayed. The significance level is .05.

Mycotoxin = Ochratoxin A

Hypothesis Test Summary

	Null Hypothesis	Test	Sig.	Decision
1	The distribution of μg/kg is the same across categories of Treatment.	Independent-Samples Kruskal-Wallis Test	.	Unable to compute.

Asymptotic significances are displayed. The significance level is .05.

Mycotoxin = T2 toxin

Hypothesis Test Summary

	Null Hypothesis	Test	Sig.	Decision
1	The distribution of μg/kg is the same across categories of Treatment.	Independent-Samples Kruskal-Wallis Test	.642	Retain the null hypothesis.

Asymptotic significances are displayed. The significance level is .05.

Mycotoxin = Zearalenone

Hypothesis Test Summary

	Null Hypothesis	Test	Sig.	Decision
1	The distribution of µg/kg is the same across categories of Treatment.	Independent-Samples Kruskal-Wallis Test	.400	Retain the null hypothesis.

Asymptotic significances are displayed. The significance level is .05.

GGraph

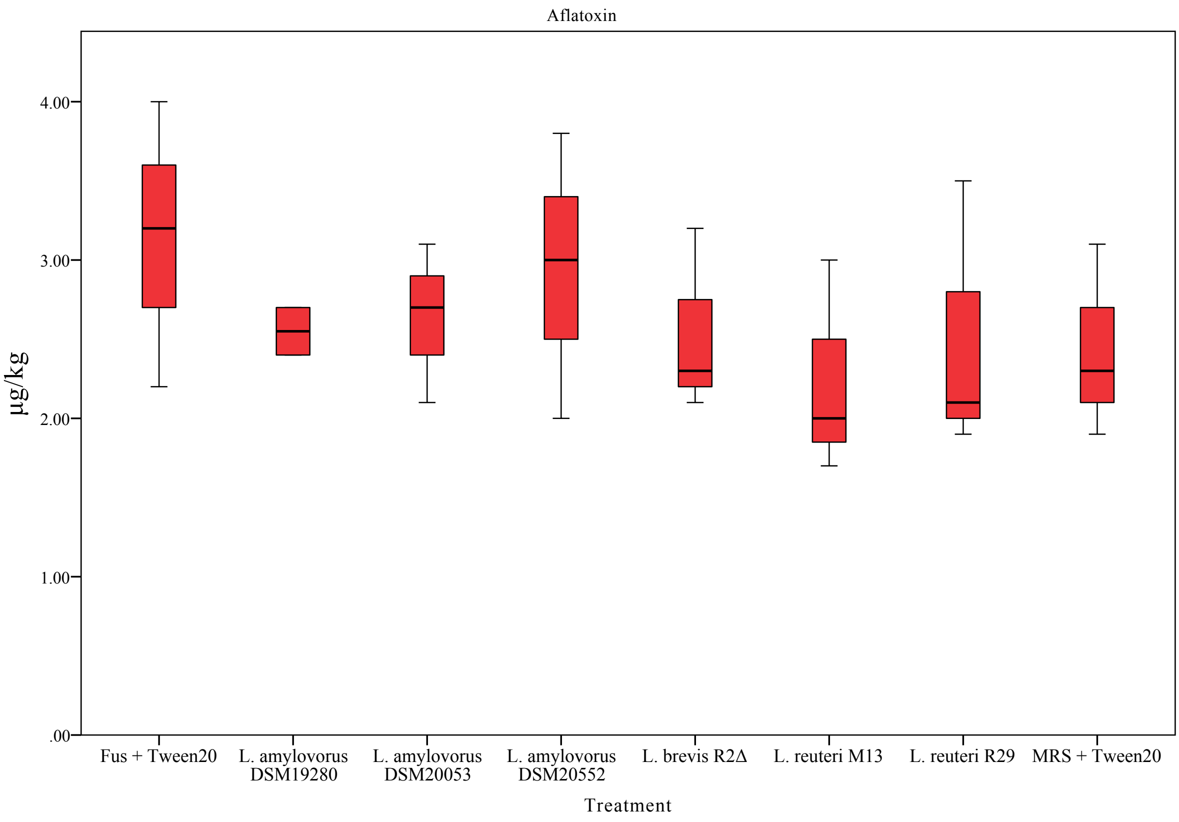

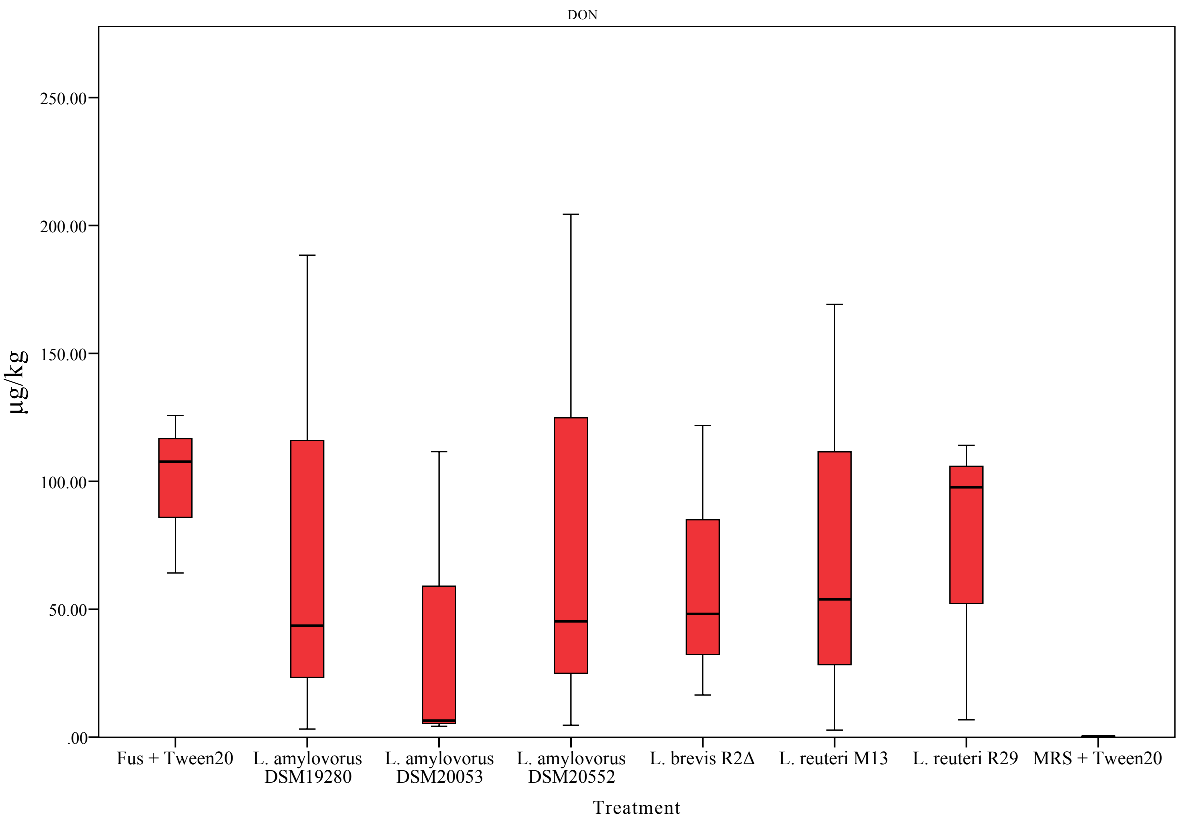
DON
250.00
200.00
150.00
100.00
50.00
.00
µg/kg
Fus + Tween20
L. amylovorus DSM19280
L. amylovorus DSM20053
L. amylovorus DSM20552
L. brevis R2Δ
L. reuteri M13
L. reuteri R29
MRS + Tween20
Treatment

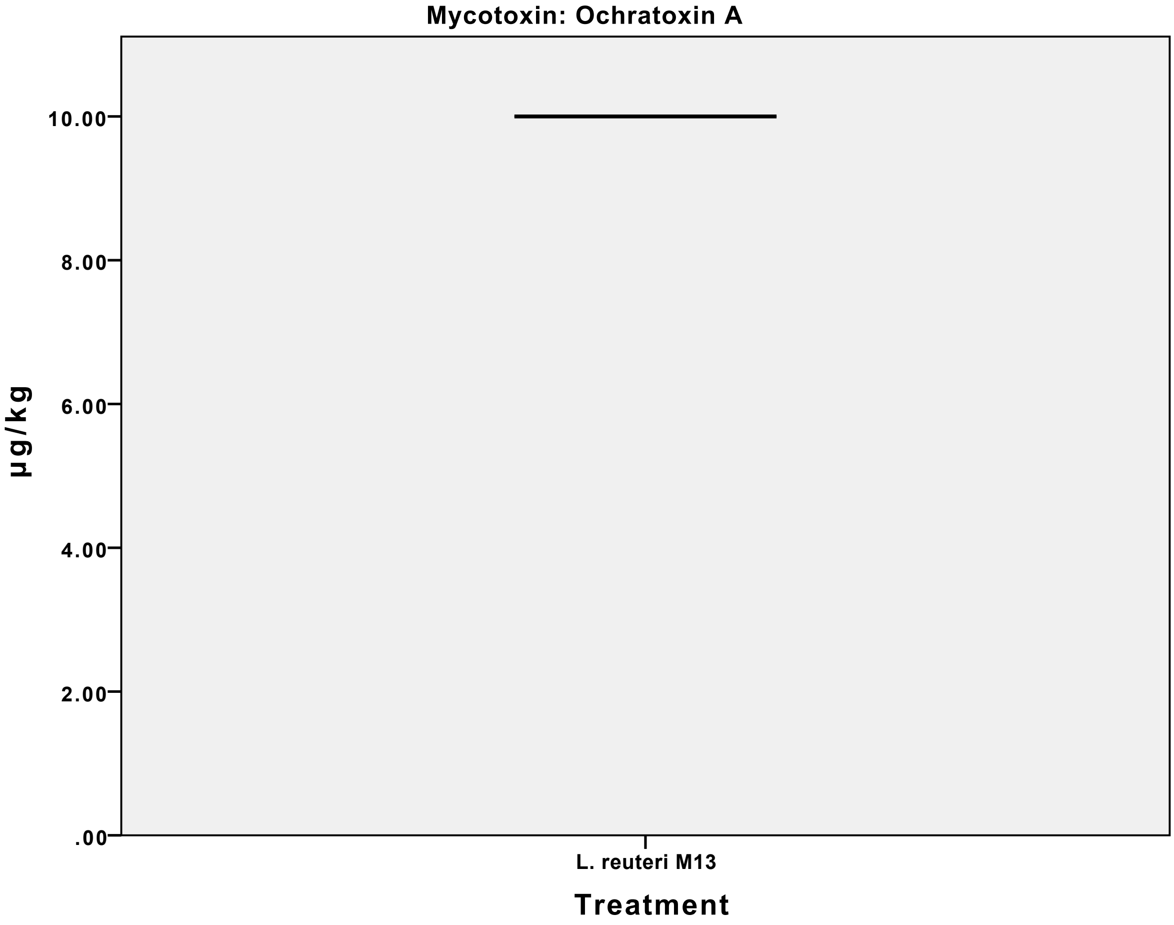

Mycotoxin: Ochratoxin A
10.00
8.00
6.00
4.00
2.00
.00
µg/kg
L. reuteri M13
Treatment

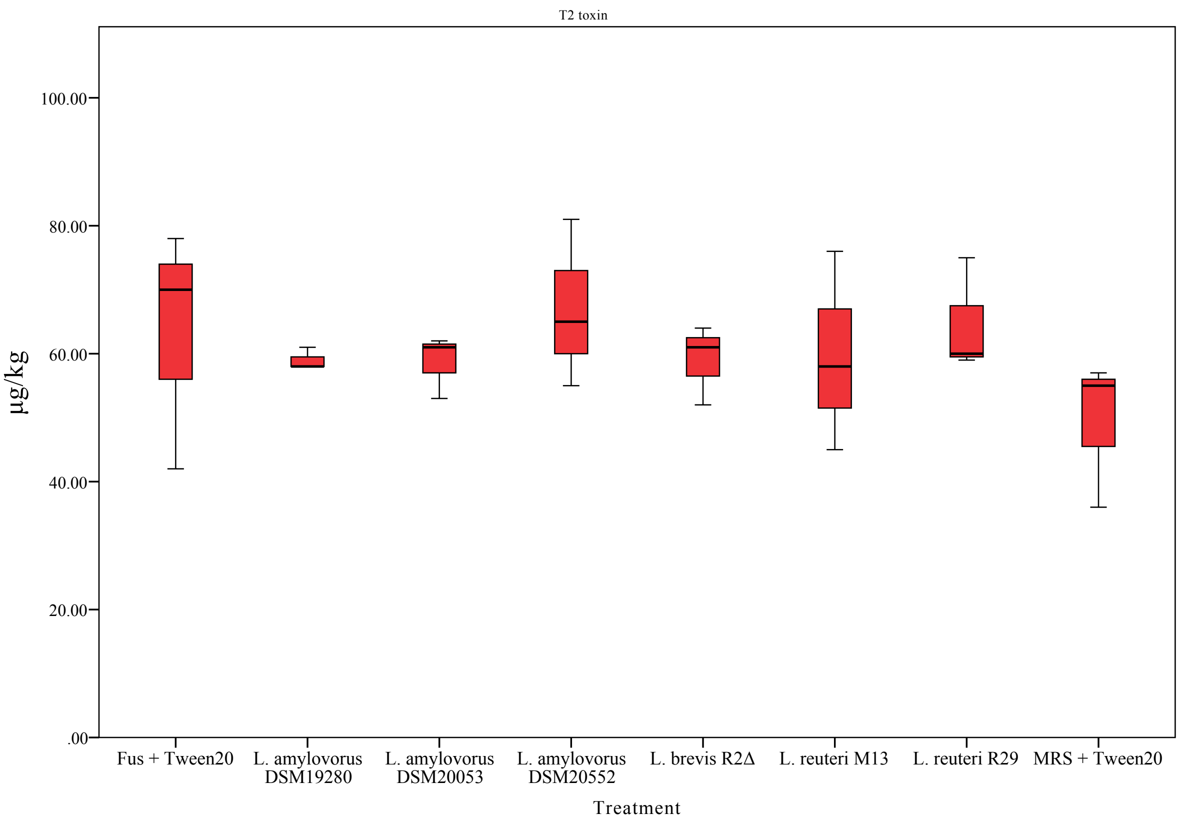

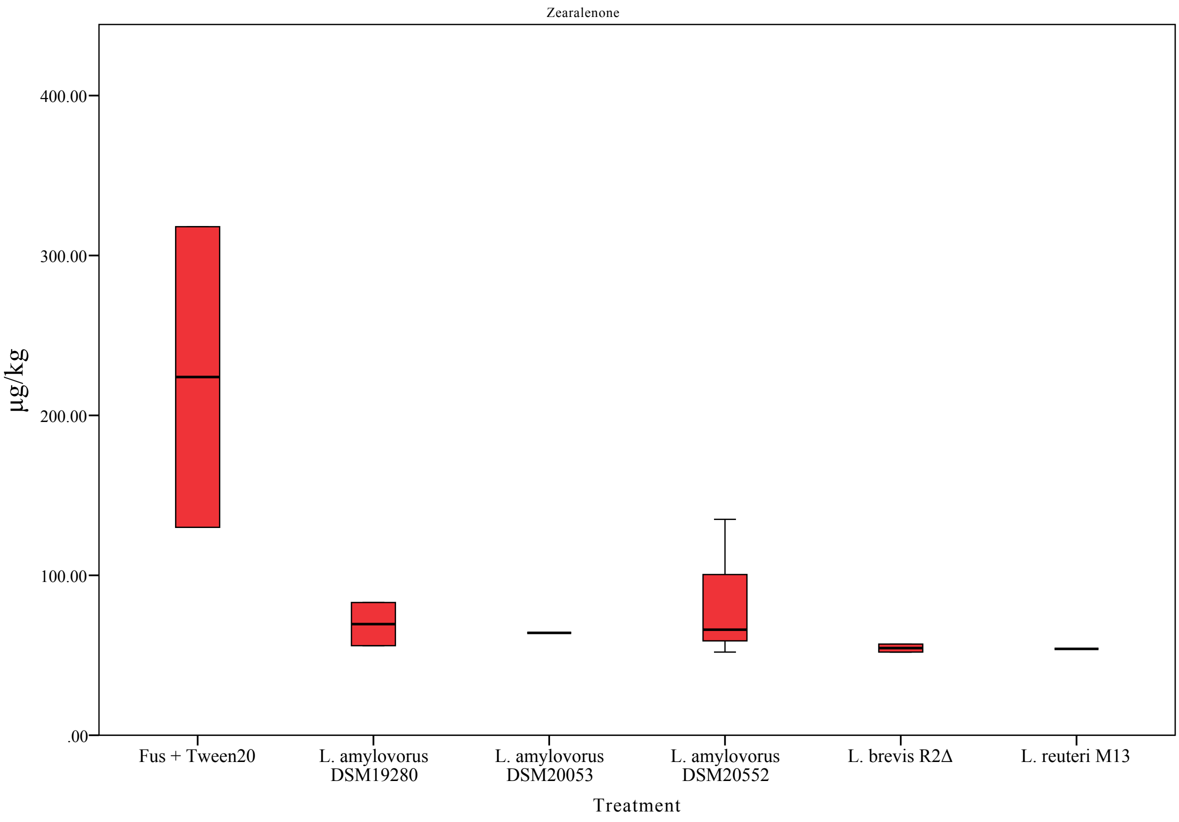

Nonparametric Correlations

Correlations

			VAR00006	VAR00007	VAR00008
Spearman's rho	VAR00006	Correlation Coefficient	1.000	.413*	.473*
		Sig. (2-tailed)	.	.045	.013
		N	30	24	27
	VAR00007	Correlation Coefficient	.413*	1.000	.784**
		Sig. (2-tailed)	.045	.	.000
		N	24	25	23
	VAR00008	Correlation Coefficient	.473*	.784**	1.000
		Sig. (2-tailed)	.013	.000	.
		N	27	23	27

*. Correlation is significant at the 0.05 level (2-tailed).

**. Correlation is significant at the 0.01 level (2-tailed).

```
 SORT CASES  BY Mycotoxin Rep.
SPLIT FILE SEPARATE BY Mycotoxin Rep.
*Nonparametric Tests: Independent Samples.
NPTESTS
  /INDEPENDENT TEST (Value) GROUP (Treatment) KRUSKAL_WALLIS(COMPARE=PAIRWI
SE)
  /MISSING SCOPE=ANALYSIS USERMISSING=EXCLUDE
  /CRITERIA ALPHA=0.05  CILEVEL=95.
```

```
 SPLIT FILE OFF.
SORT CASES  BY Rep Mycotoxin.
SPLIT FILE LAYERED BY Rep Mycotoxin.
SORT CASES  BY Rep Mycotoxin.
SPLIT FILE LAYERED BY Rep Mycotoxin.
*Nonparametric Tests: Independent Samples.
NPTESTS
  /INDEPENDENT TEST (Value) GROUP (Treatment)
  /MISSING SCOPE=ANALYSIS USERMISSING=EXCLUDE
  /CRITERIA ALPHA=0.05  CILEVEL=95.
```

Nonparametric Tests

Rep = 1, Mycotoxin = Aflatoxin

Hypothesis Test Summary

	Null Hypothesis	Test	Sig.	Decision
1	The distribution of µg/kg is the same across categories of Treatment.	Independent-Samples Kruskal-Wallis Test	.429	Retain the null hypothesis.

Asymptotic significances are displayed. The significance level is .05.

Rep = 1, Mycotoxin = DON

Hypothesis Test Summary

	Null Hypothesis	Test	Sig.	Decision
1	The distribution of µg/kg is the same across categories of Treatment.	Independent-Samples Kruskal-Wallis Test	.429	Retain the null hypothesis.

Asymptotic significances are displayed. The significance level is .05.

Rep = 1, Mycotoxin = Ochratoxin A

Hypothesis Test Summary

	Null Hypothesis	Test	Sig.	Decision
1	The distribution of µg/kg is the same across categories of Treatment.	Independent-Samples Kruskal-Wallis Test	.	Unable to compute.

Asymptotic significances are displayed. The significance level is .05.

Rep = 1, Mycotoxin = T2 toxin

Hypothesis Test Summary

	Null Hypothesis	Test	Sig.	Decision
1	The distribution of µg/kg is the same across categories of Treatment.	Independent-Samples Kruskal-Wallis Test	.429	Retain the null hypothesis.

Asymptotic significances are displayed. The significance level is .05.

Rep = 1, Mycotoxin = Zearalenone

Hypothesis Test Summary

	Null Hypothesis	Test	Sig.	Decision
1	The distribution of µg/kg is the same across categories of Treatment.	Independent-Samples Kruskal-Wallis Test	.416	Retain the null hypothesis.

Asymptotic significances are displayed. The significance level is .05.

Rep = 2, Mycotoxin = Aflatoxin

Hypothesis Test Summary

	Null Hypothesis	Test	Sig.	Decision
1	The distribution of µg/kg is the same across categories of Treatment.	Independent-Samples Kruskal-Wallis Test	.423	Retain the null hypothesis.

Asymptotic significances are displayed. The significance level is .05.

Rep = 2, Mycotoxin = DON

Hypothesis Test Summary

	Null Hypothesis	Test	Sig.	Decision
1	The distribution of µg/kg is the same across categories of Treatment.	Independent-Samples Kruskal-Wallis Test	.429	Retain the null hypothesis.

Asymptotic significances are displayed. The significance level is .05.

Rep = 2, Mycotoxin = Ochratoxin A

Hypothesis Test Summary

	Null Hypothesis	Test	Sig.	Decision
1	The distribution of µg/kg is the same across categories of Treatment.	Independent-Samples Kruskal-Wallis Test	.	Unable to compute.

Asymptotic significances are displayed. The significance level is .05.

Rep = 2, Mycotoxin = T2 toxin

Hypothesis Test Summary

	Null Hypothesis	Test	Sig.	Decision
1	The distribution of µg/kg is the same across categories of Treatment.	Independent-Samples Kruskal-Wallis Test	.429	Retain the null hypothesis.

Asymptotic significances are displayed. The significance level is .05.

Rep = 2, Mycotoxin = Zearalenone

Hypothesis Test Summary

	Null Hypothesis	Test	Sig.	Decision
1	The distribution of µg/kg is the same across categories of Treatment.	Independent-Samples Kruskal-Wallis Test	.	Unable to compute.

Asymptotic significances are displayed. The significance level is .05.

Rep = 3, Mycotoxin = Aflatoxin

Hypothesis Test Summary

	Null Hypothesis	Test	Sig.	Decision
1	The distribution of µg/kg is the same across categories of Treatment.	Independent-Samples Kruskal-Wallis Test	.429	Retain the null hypothesis.

Asymptotic significances are displayed. The significance level is .05.

Rep = 3, Mycotoxin = DON

Hypothesis Test Summary

	Null Hypothesis	Test	Sig.	Decision
1	The distribution of µg/kg is the same across categories of Treatment.	Independent-Samples Kruskal-Wallis Test	.423	Retain the null hypothesis.

Asymptotic significances are displayed. The significance level is .05.

Rep = 3, Mycotoxin = Ochratoxin A

Hypothesis Test Summary

	Null Hypothesis	Test	Sig.	Decision
1	The distribution of µg/kg is the same across categories of Treatment.	Independent-Samples Kruskal-Wallis Test	.	Unable to compute.

Asymptotic significances are displayed. The significance level is .05.

Rep = 3, Mycotoxin = T2 toxin

Hypothesis Test Summary

	Null Hypothesis	Test	Sig.	Decision
1	The distribution of µg/kg is the same across categories of Treatment.	Independent-Samples Kruskal-Wallis Test	.429	Retain the null hypothesis.

Asymptotic significances are displayed. The significance level is .05.

Rep = 3, Mycotoxin = Zearalenone

Hypothesis Test Summary

	Null Hypothesis	Test	Sig.	Decision
1	The distribution of µg/kg is the same across categories of Treatment.	Independent-Samples Kruskal-Wallis Test	.392	Retain the null hypothesis.

Asymptotic significances are displayed. The significance level is .05.
